# Promoting iron folic acid consumption using social norms as a mechanism of change in the Reduction in Anemia through Normative Innovations (RANI) project: a randomized controlled trial

**DOI:** 10.1186/s40795-025-01053-x

**Published:** 2025-04-14

**Authors:** Bee-Ah Kang, Rajiv N. Rimal, Jeffrey Bingenheimer, Rohini Ganjoo, Hagere Yilma, Erica Sedlander

**Affiliations:** 1 Department of Health, Behavior & Society, Johns Hopkins Bloomberg School of Public Health, 624 N. Broadway St. Baltimore, Baltimore, MD 21205 USA; 2 Department of Prevention and Community Health, Milken Institute School of Public Health, District of Columbia, George Washington University, Washington, USA; 3 Department of Biomedical Laboratory Sciences, School of Medicine and Health Sciences, George Washington University, Ashburn, VA USA; 4 Department of Health Sciences, Boston University Sargent College, Boston, MA USA; 5 Institute for Health and Aging, Department of Social and Behavioral Sciences, University of California, San Francisco, CA USA

**Keywords:** Social norms, Anemia, Iron, Folic acid, India, Women of reproductive age, Nutritional supplementation, Randomized controlled trial, Community-based intervention

## Abstract

**Background:**

More than 60% of women of reproductive age in Odisha, India are anemic. The national long-term efforts in reducing anemia have focused mostly on the supply side, with a paucity of campaigns on the demand side. Social norms serve as significant determinants of human behavior, but there are few interventions that adopt a social-norms approach to reducing anemia. An intervention was implemented to change descriptive, injunctive, and collective social norms to improve iron folic acid consumption behaviors among women of reproductive age.

**Methods:**

A longitudinal cluster randomized controlled trial was conducted to collect data at baseline, six months later at midline, and a year after that at end-line. All villages in our study area were formed into clusters, randomly assigned to either the treatment (50 clusters with 130 villages) or the control (39 clusters with 109 villages) arm. Women were eligible for inclusion if they aged between15 and 49, spoke Odiya, and did not plan to move in the next year. Women living in treatment communities received the intervention package that comprised community-based education sessions, health communication videos, and hemoglobin testing.

**Results:**

Data analyses included 2,061 women in the treatment arm and 2,049 women in the control arm enrolled in the trial at baseline. Hierarchical linear models revealed that all three types of social norms improved significantly more in treatment than in control communities (all *p*’s < 0.001) at midline. Two of the norms (descriptive and collective but not injunctive norms) predicted iron folic acid consumption at end-line. The relative improvement in iron folic acid consumption over time was significantly greater in treatment communities (*p* <.001).

**Conclusions:**

It appears that a social norms-based intervention can change longer-term iron and folic acid consumption behaviors to reduce anemia. Future practice may merit having norms-based strategies to promote adherence to micronutrient supplementation and medical guidelines among women. This demand-side approach will be particularly useful in resource-limited settings where the health system is inadequately prepared to procure and distribute supplements.

**Trial registration:**

This trial was registered with Clinical Trials Registry- India (CTRI) (CTRI/2018/10/016186) on 29 October 2018.

## Background

Anemia is a condition that develops when hemoglobin concentration in the blood is two standard deviations below the mean for the person’s age [1]. It affects close to half a billion women between the ages of 15 and 49, with a global prevalence of 29.6% among non-pregnant women of reproductive age and 36.5% among pregnant women [2]. Anemia results in poor cognitive and motor development in children, and it also affects their language skills [3, 4]. In adults, it reduces work capacity, enhances risks during pregnancy, and can lead to preterm births [2].

India has the world’s fifth highest prevalence of anemia among women of reproductive age [2] and is primarily caused by deficiencies in micronutrients, such as iron and Vitamin B_12_ [5, 6]. Latest data from the National Family Health Survey (NFHS-5) show that the prevalence of anemia in India has worsened since the last national survey, now standing at 57% among women of reproductive age. In the state of Odisha, prevalence is higher than the national average, at 64.3% [7]. The Government of India has spent significant resources to reduce anemia, including: promoting and distributing iron folic acid for free for pregnant and lactating women since 1970 [8]; running the Adolescent Girls Anemia Control Program that provides free supplements through the school system [9]; adopting a lifecycle approach under the National Iron Plus Initiative; and implementing the Intensified National Iron Plus Initiative (“Anemia Mukt Bharat”) since 2018 [10].

A key feature of these efforts has been the almost exclusive focus on supply-side issues, to ensure the health system is adequately prepared to acquire and distribute medications. Largely missing has been a concomitant demand-side focus to ensure that facilitators of and barriers to consumption of iron and adherence to medical guidelines are properly understood and addressed [10]. Focusing on iron-deficient anemia, with consumption of iron folic acid as the underlying outcome behavior, we ask whether a focus on social norms can serve as a theoretically informed entry point to improve behaviors and ensure their sustainability.

The study of the relationship between social norms and behaviors has a long scholarly history in a variety of disciplines [11, 12], but the conceptualization of social norms itself has tended to vary. In early social psychology research, Sherif [13] conceptualized the role of social norms as setting the social frame of reference against which one’s own judgment could be compared and contextualized, which is a perspective adopted by other influential researchers [14, 15, 16]. Sharper distinctions have emerged since those early days, with two types of norms – *descriptive norms* (to refer to people’s perceptions about others’ behaviors) and *injunctive norms* (to refer to pressures people feel to conform) [17]. Despite this focal distinction, a common thread in prevailing definitions of norms had been limited it to the realm of perceptions. Descriptive and injunctive norms all pertain to how people perceive the environment around them; it is these perceptions that, in turn, drive the underlying behavior.

Two limitations are worth noting in this conceptualization of norms. First, researchers have documented the mismatch that exists between actual behaviors and their perceived prevalence for a variety of topics – including alcohol consumption among college students [18], dietary behaviors [19], gender relationships [20], and tax compliance [21], to name a few. Perceptions need not be accurate and, indeed, research on the false consensus effect [22] and pluralistic ignorance [23] has demonstrated that they seldom are, and that media depictions further exacerbate this inaccuracy by disproportionately depicting aberrant behaviors because of their newsworthiness [24]. Interventions to change behaviors by correcting these misperceptions show mixed results [25, 26, 27, 28, 29].

The second limitation of relying solely on perceptions is that this approach neglects other important aspects of social norms, such as normative volume (the size of the prevalence and its acceptability) and normative subscription (the extent to which people endorse and internalize the norm as a behavioral guide) [24]. Lapinski and Rimal [30] proposed the idea of *collective norms* to refer to the actual prevalence of behavior and urged scholars to incorporate this concept in their theorizing [31]. In this extension, social norms are consequential in driving behaviors not only because of how people perceive those norms but also because the objective distribution of behaviors matters, regardless of how accurately they are perceived. The underlying mechanism here is less clearly understood, but one notion is that, when a behavior is highly prevalent, factors that guide its prevalence at the collective level also likely guide the behavior at the individual level.

The relationship between collective norms and individual behaviors is likely due to other factors that are not measured [30]. This idea of collective norms has since been incorporated in the extended theory of normative social behavior (TNSB) [11], which discusses the interactions across the three norms – descriptive norms, injunctive norms, and collective norms – to propose how they jointly affect behaviors [32]. This idea has taken hold in a number of studies [33, 34, 35], but the underlying causal mechanism has yet to be tested more rigorously.

In this paper, we test the causal linkages between the three normative factors, on the one hand, with iron folic acid supplement consumption, on the other. We do so in the context of a field experiment, specifically designed and implemented based on the TNSB [36]. The three innovative aspects of the work are that we (a) manipulate norms through an experimental design, (b) include collective norms as one of the normative factors that extend beyond perceptions, and (c) collect data longitudinally over the course of 18 months to test whether changes in norms from Time 1 to Time 2 drive behaviors at Time 3.

### Hypotheses

The relationship between social norms and behaviors has been tested through observational studies [37, 38], as revealed by a recent review [39]. These studies are important in understanding the underlying relationships, but they do not lend themselves to establishing causal linkages. Longitudinal studies can do so, and a number of them have been used to predict behaviors from norms over time [40, 41]. They do not, however, account for extraneous variables that confound, mediate, or moderate the relationships in complex ways.

To control for these factors, randomized studies are more robust, and findings reveal that norms are manipulable, and that they have a quantifiable impact on attitudes and behaviors, as revealed by a recent meta-analysis that included 110 articles in which norms were manipulated [42]. The fact that so many studies have manipulated social norms is, indeed, demonstrative of tremendous progress in the scholarship on social norms. However questions about the durability of norms-induced behavioral outcomes can also be addressed going beyond short-term changes in a laboratory setting, as pointed out by scholars [43]. We answer that call in this paper by manipulating norms in a field experiment and assessing their impact on iron folic acid consumption over 18 months.

We draw on the three social norms constructs (descriptive, injunctive, and collective norms), hypothesizing that the intervention resulted in improvements in each of these norms from Time 1 to six months later at Time 2. Second, we expect that changes in norms from Time 1 to Time 2 to predict iron folic acid consumption (behavioral outcomes) at Time 3 (a year after Time 2), after controlling for the Time 1 consumption behavior. Third, we also expect that, overall, there were significant improvements in behaviors after the intervention. Hence, our hypotheses are:

H1: From Time 1 to Time 2, improvements in descriptive norms (H1a), injunctive norms (H1b), and collective norms (H1c) will be greater in treatment than in control communities.

H2: Improvements in descriptive (H2a), injunctive (H2b), and collective (H2c) norms from Time 1 to Time 2 will predict iron folic acid consumption at Time 3, after controlling for the behavior at Time 1.

### Methods

Data for this study come from the Reduction in Anemia through Normative Innovations (RANI) Project, a field experiment run in the state of Odisha in eastern India over an 18-month period [44]. The primary aim of the study was to determine whether a social norms-based intervention could reduce anemia among women of reproductive age by promoting the consumption of iron folic acid tablets according to the World Health Organization guidelines (once per week for nonpregnant women and daily for pregnant women). Informed by the TNSB [36], the intervention itself was developed based on findings from a six-month long, mixed methods formative evaluation which included 16 focus groups and 21 individual interviews with stakeholders [45] which sought to understand the role that gender and other aspects of self-identity played in women’s decisions to take iron supplements, and the extent to which their mothers-in-law, husbands, and other community members could be mobilized to facilitate women’s iron consumption.

### Development of the RANI intervention

A key finding from the qualitative formative assessment [10, 46] was that the drivers of taking iron folic acid tablets resided at multiple levels in the socioecological continuum. Apart from individual level factors, such as perceived side effects and low risk perceptions, drivers of the behaviors were also manifest at the interpersonal (how husbands and other influential people perceived the importance of anemia), structural (having access to the tablets), and cultural (the importance of prioritizing the health of women in the community) levels.

An outcome from this work was the tentative affirmation of the team’s idea that a social norms-based approach was probably an effective strategy for a longer-term behavior change approach. Over a week-long participatory workshop, this proposed approach was then shared with stakeholders, which included members from the local *Panchayat* (political leadership), leaders from women’s self-help groups (which have a long-standing engagement in the community), frontline healthcare workers (in Odisha, they are the *Aganwadi* workers, accredited social health activists [also called ASHAs], and auxiliary nurse midwives [also called *ANMs*]), community residents themselves, and members of nongovernmental organizations engaged in nutrition activities in the state. During the workshop, we shared our findings from the formative assessment, discussed the role of social norms, and collectively co-designed and subsequently refined a few social norms-based approaches.

Because the key elements of the RANI intervention are published elsewhere [47], we do not describe them again here, except to note that activities were specifically designed to change: *descriptive norms* (by highlighting the fact that more and more women of reproductive age were now consuming iron folic acid as part of a healthy behavior); *injunctive norms* (by enlisting the support of husbands, mothers-in-law, community leaders, and the health system to facilitate, prioritize, and promote the distribution of iron folic acid for free to all women of reproductive age in the community); and *collective norms* (by displaying in community centers and other prominent locations the increasing hemoglobin levels, which are measures of improvements in anemia, in the community through graphically-based results).

To promote social and behavior change, we engaged the community in participatory learning modules through hands-on activities [47, 48], and we also developed and disseminated six videos that provided education about the link between iron and anemia and role modeled people who were supporting women in their consumption of iron supplements. The videos targeted our focal populations (adolescent girls and married women of reproductive age) as well as other influential people in their social networks, including husbands, mothers-in-law, and community members. The underlying idea, based on the TNSB, was that the appeal to change needed to be made to both parties – the target audiences themselves and their social network members. This is what sets apart a social norms-based intervention from an individual-focused intervention. Whereas an individual-focused intervention makes the case for change directly to the target audience, a social norms-based intervention also includes an appeal that is made to influential others, whose opinions would be consequential in the target audience members’ decisions about whether to enact the recommended behavior. For these reasons, we showed the videos not only to the target audience (for whom the specific video was produced) but also to their influential parties – so that our target audience members would know that others had seen the video and that those others were also expected to support the target audience members in their decisions to consume iron supplements. These videos were shown in small groups, and they were also screened publicly for residents during community events.

### Study design

The evaluation design consisted of a three-wave longitudinal cluster randomized trial in which we collected data from the same women at baseline (Time 1) in September 2019, six months later at midline (Time 2) in February 2020, and a year after that at end-line (Time 3) in August 2020. At each wave, we conducted one-on-one interviews (approximately an hour in duration), obtained women’s height and weight measurements, and assessed psychosocial variables. The same procedures were adopted in both treatment and control communities.

**Fig. 1:**
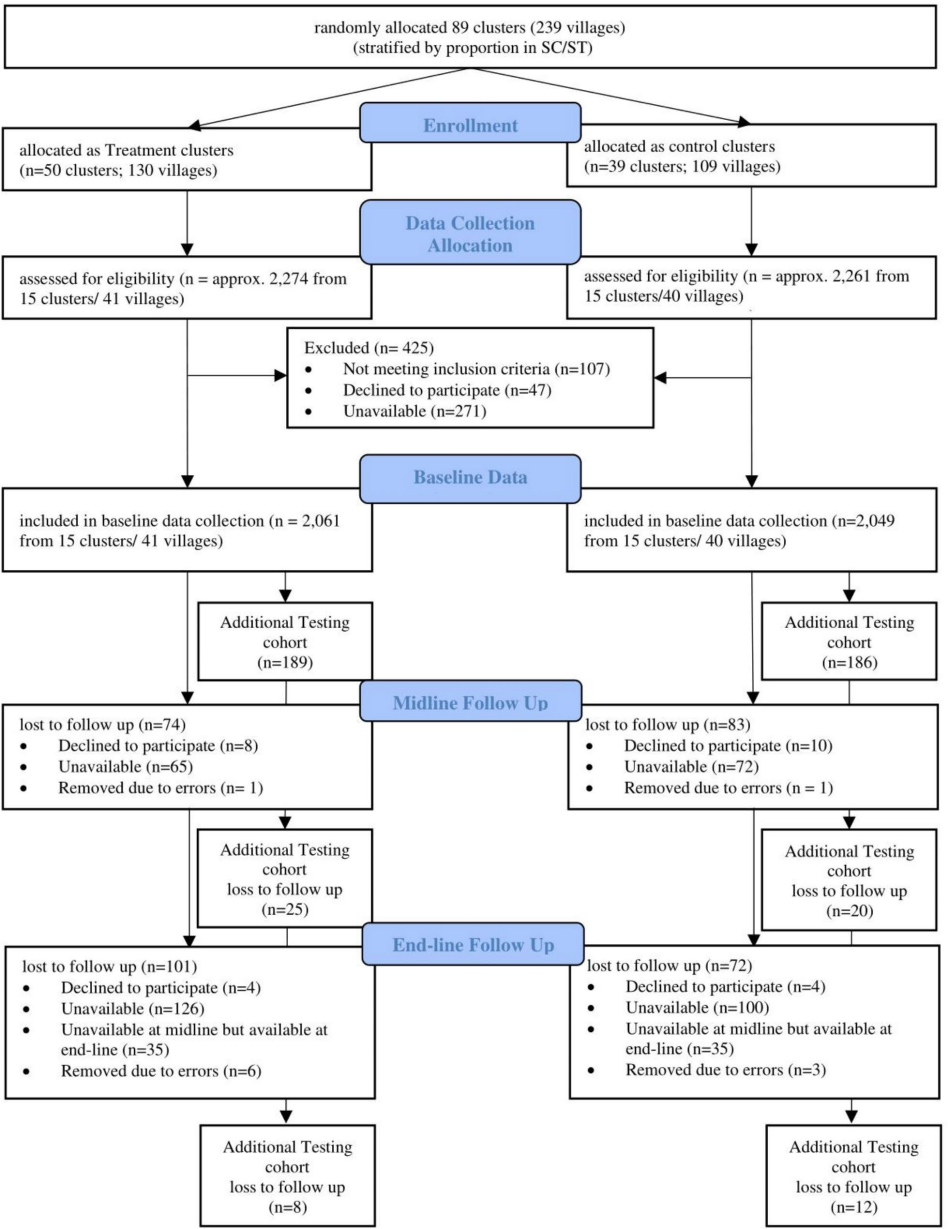
Flow chart of the RANI project

Randomization into treatment and control communities was done in three stages (Fig. 1). First, all villages in our study area (*k* = 239) were formed into 89 clusters, with each cluster including two to four geographically contiguous villages. Second, all adjacent clusters were excluded from sampling in order to minimize contamination. Third, all remaining clusters (which now had a buffer of at least one or more contiguous clusters) were randomly assigned to either the treatment (50 clusters that comprised 130 villages) or the control (39 clusters with 109 villages) arm.

Within each cluster, villages were stratified by their concentration of tribal populations (to ensure we captured a sufficient number in our study) and then homes were sampled in proportion to population size. In each selected home, we invited one woman of reproductive age to participate in the study (randomly selecting from a list if more than one eligible woman resided in the home). Women were considered eligible if they were between 15 and 49 years old, a resident of the village in Odisha, spoke Odiya, and did not have a plan to move out of the village for the next two years.

Baseline data were collected from all participants before the start of the intervention. In the control arm, we only collected the three waves of data, without any intervention activities. In the treatment arm, the same procedures were adopted for data collection, with the intervention being rolled out immediately after the baseline data collection was completed. At baseline, interviewers were blinded about the treatment or control status of the village from which participants were recruited; although the treatment/control status was never revealed to data collectors, it is likely that they came to learn about the status after conducting a few interviews at midline. On the research team, only the statistician knew the treatment or control status of any given participant; the principal investigator and others were shielded from this information through a password-protected file.

Informed consent was obtained by data collectors before beginning the interview. Participants signed a form to indicate their consent. Ethics approvals were obtained from the Institutional Review Board at George Washington University in Washington DC, USA (FWA00005945), the Institutional Review Board at Sigma Science and Research in New Delhi, India (10031/IRB/D/18–19 for baseline 10059/IRB.19–20 for midline, and 10036/IRB20-21 for end-line), and from the India Council for Medical Research (ICMR)’s Health Ministry Screening Committee (HMSC) (2018 − 0921/F1).

### Measurement

All variables used in this study were obtained through one-on-one interviews conducted by Oriya-speaking women interviewers who had received a weeklong human subjects and interviewing training.

#### Demographic measures

We measured *age* in years, *education* as the number of years spent in school, *tribal status* as whether the participant belonged to one of the tribes recognized by the government, whether the participant was *pregnant* at the time of data collection, and the *number of children* she had.

#### Descriptive norms

Informed by the TNSB [36], we assessed descriptive norms as the average of three questions that asked participants to estimate what proportion of women in their communities took iron folic acid tablets. Responses, measured on five-point scales (from 0 to 4) were averaged into an index α = 0.45 at baseline and α = 0.63.

#### Injunctive norms

Following the TNSB [36], we assessed injunctive norms through six questions by asking participants how many others in their social network (including other women in their community, their mother-in-law, husband) thought the participant should take iron folic acid. Responses, coded on five-point scales (from 0 to 4) were averaged into an index, α = 0.71, α = 0.77 at midline.

#### Collective norms

Following the method proposed by Sedlander and Rimal [49], we assessed collective norms as the “non-self mean”–by calculating the total iron folic acid consumption in the village (based on everyone from the village in our sample), subtracting out the consumption level of the participant, and then dividing this difference by the total number of women from the village in the sample, minus one. Numerically, the collective norm measure is:

$$\:collective\:norms=\:\frac{\left[\sum\:_{k=1}^{k}\left(IFA\right)\right]-\left({IFA}_{k}\right)}{(k-1)}$$

where *k* = number of participants in the given village in our data set and *IFA* = whether each person reported consuming iron folic acid (coded as 1) or not (coded as 0).

Iron folic acid consumption was asked through a single item, whether the participant had ever taken iron folic acid in the past, with three response options: never taken in the past, taken in the past but not taking now, and taking now. The last option was coded as 1 and the first two options (never consumed or only prior consumed) was coded as 0.

#### Change scores

Change scores in the three (descriptive, injunctive, and collective) norms were calculated by subtracting baseline values from the corresponding end-line values of the norms.

### Statistical tests

Due to the nested nature of the data, we used hierarchical linear models whenever appropriate; in longitudinal comparisons, individuals were treated as Level 2 and clusters as Level 3 in mixed effects regressions.

### Ethical considerations

This trial is registered with the Clinical Trial Registry of India (CTRI/2018/10/016186).

## Results

### Attrition and differential attrition

At baseline, we recruited 2,049 women from control and 2,061 women from treatment communities for a total of *N* = 4,110. At end-line, those remaining in the sample included *N* = 1,894 in control and *N* = 1,886 in treatment arms. This corresponded to attrition rates of 7.5% and 8.4% in control and treatment arms, respectively, and the test for differential attrition showed it was not significant *X*^2^ = 1.20, *p* >.05.

We did find that those who remained in the sample at end-line (*M* = 30.7, *SD* = 8.73) were older (*t* = 11.28, *p* <.001) than those who dropped out (*M* = 25.12, *SD* = 7.85); those who remained (*M* = 6.12 years of schooling, *SD* = 4.10) were also less educated (*t* = 8.08, *p* <.001) than those who dropped out (*M* = 8.02 years of schooling, *SD* = 4.10). For tribal status and pregnancy status – those who dropped out were not statistically different from those who remained in the sample.

### Description of the sample

Table 1 shows the description of the sample, in both control (*N* = 2,049) and treatment (*N* = 2,061) communities. A few things to note are that the sample consisted predominantly of women between 21 and 40 years old (68% in the control and treatment communities). Roughly 40% of the sample in both communities had up to a tenth-grade education, with approximately 18% of the women having received no formal education at all. Proportion of women who were pregnant at baseline was 5.2% in control and 4.4% in treatment communities. At baseline, because of the randomization, none of the demographic variables differed significantly between the control and treatment communities.

**Table 1:** Characteristics of the sample at baseline in control and treatment communities

Control (*n* = 2,049)	Treatment (*n* = 2,061)	Chi-sq.	*F*-value
	*M* (*SD*) %	*M* (*SD*) %	
Age			
15–20 years	16.8	16.0	
21–30 years	38.7	37.0	
31–40 years	29.7	31.3	
41–50 years	14.8	15.8	2.30
Education			
None	17.9	18.6	
Up to 6th grade	28.2	26.7	
Up to 10th grade	40.4	42	
Up to 12th grade	10.1 9.1	9.1	
> 12th grade	3.5	3.5	4.30
Member of a tribe	31.4	24.8	0.92
Pregnant	5.2	4.4	2.08
Number of children	1.61 (1.28)	1.64 (1.23)	0.43

Note. None of the variables differed between control and treatment communities at baseline

### Test of hypotheses H1a, H1b, and H1c

Hypotheses H1a, H1b, and H1c predicted that, from baseline to midline, the RANI intervention would result in improvements in descriptive, injunctive, and collective norms, respectively. Table 2 shows that, from baseline to midline, all three norms improved significantly more in treatment than in control communities. Descriptive norms improved in the control communities from *M* = 1.08 (*SD* = 0.62) to *M* = 1.23 (*SD* = 0.62), whereas the corresponding improvement in the treatment communities was from *M* = 1.07 (*SD* = 0.60) to *M* = 2.06 (*SD* = 0.67), which was significantly greater than the corresponding improvement in control communities, *z* = 33.43, *p* <.001.

**Table 2:** Changes in norms in control and treatment communities from baseline to midline from hierarchical linear models (HLM)

		Baseline		Midline		*Z*-value^a^
Type of Norm		Control	Treatment	Control	Treatment	
		(*n* = 2,049)	(*n* = 1,966)	(*n* = 1,987)		
Descriptive						33.43***
	*M*	1.08	1.07	1.23	2.06	
	*SD*	0.62	0.60	0.62	0.67	
Injunctive						27.68***
	*M*	2.66	2.55	2.92	3.60	
	*SD*	0.74	0.74	0.75	0.59	
Collective						174.66***
	*M*	0.06	0.05	0.05	0.33	
	*SD*	0.04	0.03	0.04	0.12	

^a^*Z-*test compares the improvement in norms from baseline to midline in control communities against a similar improvement in treatment communities. For all three norms, improvements in treatment communities were significantly greater than in control communities. Results are based on mixed effects regressions with random effects of individuals at Level 2 and randomization clusters at Level 3

***p <.001

Similarly, injunctive norms improved in the control communities from *M* = 2.66 (*SD* = 0.74) to *M* = 2.92 (*SD* = 0.75), whereas the corresponding improvement in the treatment communities was from *M* = 2.55 (*SD* = 0.74) to *M* = 3.60 (*SD* = 0.59), which was significantly greater than the improvement in control communities, *z* = 27.68, *p* <.001.

Collective norms also improved substantially in treatment communities – from *M* = 0.05 (*SD* = 0.04) at baseline to *M* = 0.33 (*SD* = 0.12) – as compared to control communities, where collective norms decreased – from *M* = 0.06 (*SD* = 0.04) at baseline to *M* = 0.05 (*SD* = 0.04 at midline). The relative improvement in treatment communities was significantly greater than in control communities, *z* = 174.66, *p* <.001.

Thus, we concluded that the RANI intervention significantly improved descriptive norms, injunctive norms, and collective norms. This supports Hypotheses H1a, H1b, and H1c.

### Tests of hypotheses H2a, H2b, and H2c

Hypothesis H2a through H2c proposed that the intervention led improvements in the three norms would, in turn, affect improvements in iron folic acid consumption. To test this hypothesis, we used the three variables that captured improvements in descriptive norms, injunctive norms, and collective norms from baseline to midline (calculated as the difference between midline and baseline values) to predict iron folic acid consumption at end-line, running these analyses separately in control communities (where only secular trend-driven changes would be in play) and in treatment communities (where the norms themselves were being changed by the intervention). We used logistic regressions, with end-line iron folic acid consumption status (either taking, coded as 1, or not taking, coded as 0) as the dependent variable, controlling for baseline iron folic acid consumption status and pregnancy at each of the two waves (because iron folic acid uptake is promoted by the health system during pregnancy). Predictors were change scores from baseline to midline in each of the three social norms.

Baseline iron folic acid consumption (measured at the beginning of the intervention) predicted end-line consumption (measured 18 months later) – adjusted odds ratio (AOR) = 3.99, *p* <.05, signifying that those who consumed iron folic acid at baseline were four times more likely to do so at end-line (Table 3). This relationship was not significant in treatment communities.

**Table 3:** Predictors of iron folic acid consumption at end-line in treatment and control communities from multilevel logistic regressions

		Control Communities				Treatment Communities		
		(*n* = 1,849)				(*n* = 1,851)		
	B	RSE	AOR	RCI	B	RSE	AOR	RCI
Baseline consumption	1.38*	0.56	3.99	(1.33, 12.01)	-0.44	0.52	0.64	(0.23, 1.80)
Pregnant at baseline	-0.56	0.80	0.57	(.12, 2.74)	0.52	0.67	1.68	(0.45, 6.30)
Pregnant at midline	3.86***	0.37	47.67	(23.00, 98.80)	-0.23	0.35	0.79	(0.40, 1.57)
∆Descriptive norms	-0.1	-0.1	.10.90	(0.75, 1.09)	.26*	0.12	1.29	(1.02, 1.66)
∆Injunctive norms	-0.02	0.1	0.99	(0.80, 1.19)	0.06	0.15	1.06	(0.79, 1.44)
∆Collective norms	2.74	2.81	15.49	(0.06, 3850.17)	2.31*	1.07	10.08	(1.24, 81.81)

Notes. B = beta coefficient from logistic regression; RSE = robust standard error; AOR = adjusted odds ratio; RCI = robust confidence interval. Changes in the three norms are improvement at midline, compared to baseline. Baseline = Month 1, Midline = Month 7, End-line = Month 19

*p <.05, **p <.01, ***p <.001

Pregnancy at baseline was not associated with iron folic acid consumption at end-line, but pregnancy status at midline was positively associated with iron folic acid consumption at end-line – though only for women in control communities.

None of the three changes in social norms was associated with iron folic acid consumption in control communities. In treatment communities, however, improvement in descriptive norms was significantly associated with consumption (AOR = 1.29, *p* <.05) as was improvement in collective norms (AOR = 10.08, *p* <.05). Improvement in injunctive norms was not associated with consumption. Hence, Hypothesis H2 found support for two of the three norms in treatment communities, but not in control communities.

Table 4 shows the percentages of iron folic acid consumption at baseline and end-line by study arm. The relative improvement in iron folic acid consumption over time was significantly greater in treatment communities than in control communities, *z* = 83.21, *p* <.001. Therefore, we concluded that the RANI intervention was effective in promoting behavior change for anemia reduction.

**Table 4:** Iron folic acid consumption in control and treatment communities at baseline and end-line

		Baseline		Endline		*Z*-value^a^
		Control	Treatment	Control	Treatment	
(sample size, *n*→)		(2049)	(2061)	(1,894)	(1,886)	83.21***
Iron folic acid consumption		6.10%	5.40%	6.80%	93.20%	

Note. ^a^*Z*-test compares improvement in iron folic acid consumption from baseline to end-line in control communities with similar improvement in treatment communities. Results are based on mixed effects regressions with random effects of individuals at Level 2 and randomization clusters at Level 3

***p <.001

## Discussion

The primary objectives of this paper were two-fold: to assess the extent to which social norms could be improved through an intervention and whether those improvements would lead to improved iron folic acid supplement consumption. Specifically, we tested two propositions: (1) whether the RANI intervention changed descriptive, injunctive, and collective norms, and (2) whether changes in norms affected iron folic acid consumption behaviors. We found significant evidence for these propositions.

A key feature of this paper was the finding that interventions can bring about changes in all three types of norms. Descriptive norms, for example, were equivalent and low in control and treatment communities before the start of the intervention (1.08 and 1.07, respectively). By the end of the six-month period at midline, however, descriptive norms had improved by almost two-fold in treatment communities, whereas their improvement in control communities was only about 23%. We saw similar patterns of greater improvement in treatment, compared to control communities for the other two norms as well: improvement in injunctive norms in control communities was approximately 10% but that in treatment communities was approximately 41%. The largest jump we saw was an improvement in collective norms – it decreased from 0.06 to 0.05 in control communities, but it improved by more than 400% in treatment communities.

We suspect some of the dramatic improvement in collective norms was due to social desirability. Collective norms, after all, are based on peoples’ self-reported consumption of iron folic acid, which the campaign was promoting in treatment communities (without such efforts in control communities). Hence, it is likely that participants inflated their reporting of the very behavior they knew the campaign was promoting. For this reason, we are somewhat skeptical about the magnitude of the four-fold increase in collective norms. This skepticism, however, has to be modulated on the basis of the other findings: It is difficult to see why people would also provide an inflated estimate of others’ behaviors (to affect heightened descriptive norms) or pressures on them to conform (to account for heightened injunctive norms) if it were all driven by social desirability biases. Indeed, if participants themselves were not consuming iron folic acid, pressures on reporting the consumption pattern of others would likely have operated in the opposite direction – to report that others, too, were not consuming, rather than inflating others’ consumption. Hence, a more plausible conclusion is that the three norms likely improved because of the RANI intervention, but the actual magnitude of the increase may be less than what we report.

The underlying mechanism of change is illustrated in Table 3. We note first that, in the absence of an intervention, consumption of iron folic acid at baseline predicted consumption at end-line in the control communities, but this was not the case in treatment communities. This is the first illustration of the finding that the RANI intervention disturbed the homeostatic balance: in its absence, behaviors were mostly habitual (prior behaviors predicted future behaviors), but prior behaviors did not predict future ones when an intervention was actively promoting change.

Improvements in descriptive and collective norms in treatment communities six months into the intervention predicted improvement in iron folic acid consumption a year after that; this was not the case in control communities. Improvements in injunctive norms, however, did not predict iron folic acid consumption. One reason why only two of the three norms predicted behaviors may have to do with the magnitude of change: among the three normative factors shown in Table 2, injunctive norms had the lowest level of improvement, as compared to improvements in descriptive norms and collective norms. Our finding that injunctive norms did not influence behaviors is at odds with the review of the meta-analysis reported by Rhodes, Shulman, and McClaran [42], and one possible explanation is that our improvement in injunctive norms was not large enough to drive behavioral change. This is worthy of future exploration.

We should also note that pregnancy at baseline did not predict iron folic acid consumption in either control or treatment communities. Midline pregnancy status, however, predicted iron folic acid consumption at end-line in control but not in treatment communities. Pregnancy status was an important variable to consider, for two reasons. First, in much of India, as soon as a pregnancy is registered with the healthcare system, the woman’s anemia status and other pregnancy-related risk factors are supposed to be closely monitored by frontline healthcare workers. The woman is counseled to attend prenatal visits, where her vitals are monitored and where she receives iron folic acid tablets for free. Second, the recommended frequency of iron folic acid consumption for pregnant women is one tablet per day, whereas that for nonpregnant women is one tablet per week.

That pregnancy status at midline predicted consumption at end-line – but only in control communities – likely speaks to the fact that pregnant women in control communities were being monitored and provided free iron folic acid tablets, leading to higher rates of consumption. In this way, it was pregnancy that was driving consumption and distinguishing women who were taking iron folic acid from those who were not. In treatment communities, however, the situation was a bit different: what distinguished consumption was not pregnancy status but the fact that all women were being subjected to an external intervention. Put another way, the RANI intervention’s promotion of iron folic acid among all women erased the salience of pregnancy status as the primary driver of consumption (as was seen in control communities).

Finally, we note that the overall intervention was fairly intensive in that it contained many components, delivered over a significant period of time. While having an intensive intervention improves the odds of finding an impact, the trade-off, of course, is that such interventions become less scalable. In this study, we opted for a more intensive intervention, given that social norms-based interventions to reduce anemia had not been tried in a field setting, and we wanted to investigate whether norms could be changed and whether behavior change would ensue in a field setting like ours. A component analysis in the future could inquire about which elements of the intervention maximize both impact and scalability.

### Limitations

Some measures (particularly descriptive norms) suffered from low reliability, indicating the presence of significant error variance, even though the measures we used were informed by prior work done under the auspices of the theory of normative social behavior [36]. Nevertheless, the fact that indexes with low reliability scores still predicted behavior at a subsequent time indicates that, had we included more robust measures, our effect sizes may have been slightly larger.

Perhaps the greatest threat to validity lies in the self-report measures we used to assess most of our variables. This is especially worrisome in a study design like ours in which no interactions occurred between community residents and the study team during the 18-month period in the control arm, whereas the opposite was true in the treatment arm. Implementing our intervention activities necessarily required the presence of our study team on the ground, with regular and extensive interactions with community residents over the study period. Hence, the control and treatment arms differed not only in the nature of the intervention – the norms-based approach, learning activities, communication messaging – but also in terms of the protracted engagement with community members that took place in treatment but not in control communities. This level of engagement likely resulted in treatment community residents developing stronger relationships with and a sense of obligation toward the RANI project personnel. This could have led to respondents providing the study team with socially desirable responses. Inclusion of other measures pertaining to descriptive and injunctive norms likely reduced this bias.

We also note that those who remained in our longitudinal sample were significantly older and less educated, compared to those who dropped out. This also compromises our external validity. We note, however, that the older and less-well educated women are likely those for whom an intervention like ours would be more relevant, as they would represent an older generation with fewer options for self-care and social mobility, compared to those who are younger and better educated, as has been found in China and India [8]. Overall, our large, blinded cluster-randomized control trial design ensured applicability, and we expect our findings to be generalizable to other clusters in India or to women of reproductive age with high iron demands.

## Conclusions

The RANI Project was designed to improve descriptive, injunctive, and collective norms pertaining to the consumption of iron folic acid to reduce anemia among women of reproductive age in Odisha, India. Over the 18-month intervention period, the cluster randomized trial found significant improvements in all three norms. Improvements in descriptive and collective norms, but not injunctive norms, from baseline to midline six months later predicted iron folic acid consumption at end-line, 12 months after midline.

## Data Availability

Data are available in a public, open access repository. Our data can be found in the GWU Scholar Space online repository (https://scholarspace.library.gwu.edu/) and by request from the first author.
